# A validated workflow for the design and certification of below-the-hook lifting devices integrating finite element analysis and physical proof testing

**DOI:** 10.1038/s41598-025-31175-y

**Published:** 2025-12-17

**Authors:** El-Sayed Habib, Eslam Shamso

**Affiliations:** 1 Mechanical Engineering Department, Faculty of Engineering, Suez University, Suez, 43512 Egypt; 2 Production Engineering and Mechanical Design Department, Port-Said University, Port-Fouad, 42523 Egypt

**Keywords:** Spreader beam, FEA analysis, Structural design, Proof load test, ASME BTH-1, Non-Destructive testing (NDT), Engineering, Materials science

## Abstract

This paper presents a comprehensive, standardized workflow for the design, analysis, fabrication, and validation of a 30-ton capacity spreader beam as a case study. The methodology integrates computational engineering with rigorous physical testing to ensure compliance with international safety standards, including ASME BTH-1 and OSHA. The process begins with defining requirements and proceeds through preliminary design, material selection, and detailed Finite Element Analysis (FEA) using Ansys. An iterative approach was employed to optimize the design, transitioning from an initial 6-inch pipe concept to a finalized 8-inch ASTM A106 Grade B pipe main beam with ST37 plate lugs. The optimized design achieved a safety factor exceeding 2.0, with a maximum von Mises stress below 100 MPa. The physical prototype was fabricated under strict quality control, including weld inspection via Liquid Penetrant (PT) and Magnetic Particle Testing (MT). Final validation was achieved through a successful 40-ton (133% of Safe Working Load) proof load test. The beam was subsequently certified for operational use, with recalibration required every six months. This work provides a validated, step-by-step guide for engineers, demonstrating a critical balance between analytical prediction and experimental verification in lifting equipment design.

## Introduction

The safe and efficient lifting of heavy loads is a critical operation in industries ranging from construction and energy to logistics and manufacturing. Spreader beams are indispensable lifting devices designed to convert lifting loads into pure compression, thereby preventing damage to slings and cargo by enabling balanced, multi-point lifts from a single crane hook1. The fundamental design principles for such equipment, including load calculations and structural configurations, are well-established in foundational literature^1^.

The advent of Computer-Aided Engineering (CAE) has revolutionized the design process for lifting equipment, moving it from traditional, often conservative, manual calculations towards optimized, validated digital prototypes. Finite Element Analysis (FEA) is now a cornerstone of this process, allowing for precise prediction of stress distribution, identification of concentration points, and virtual optimization before physical fabrication^2^. The capability of FEA software like ANSYS and COMSOL to ensure structural safety and efficiency is well-established, with ANSYS frequently applied in mechanical component design^3,4^. and COMSOL excelling in solving coupled multi-physics problems^2,5^.

This capability is clearly demonstrated in the domain of lifting equipment. For instance, Kusumaa et al.^6^ utilized FEA in SolidWorks to design and optimize an 80-ton spreader bar, evaluating multiple lifting configurations to ensure a safety factor of 1.5, as employed in their study, was met. Similarly, Gopagoni and Kumar^7^ relied on ANSYS to perform crucial structural analysis on a 350-ton capacity lifting beam under both symmetric and asymmetric loading, underscoring the method’s critical role in preventing failure.

The value of FEA extends beyond mere compliance; it enables true design optimization. This is evidenced by studies such as İrsel^8^, whose integrated CAE and experimental validation approach for agricultural machinery led to a significant 35.85 kg mass reduction while maintaining structural integrity. The critical role of detailed component design is further highlighted by Dhage et al.^9^, who used FEA to optimize the cross-sectional geometry of lifting eye-bolts, demonstrating a 24% stress reduction by moving from a ring to a trapezoidal design. Furthermore, advanced computational techniques are being applied to enhance performance in related domains; for instance, topology optimization has been successfully used to minimize the volume of lifting hooks while maintaining strength^10^, and combined experimental-FEA methods have been employed to significantly improve the energy absorption of thin-walled structures through trigger mechanisms and heat treatment^11^. The push towards automation in design selection, exemplified by Wheatley et al.’s^12^ algorithm for modular spreader beams, highlights the next frontier in engineering efficiency. This study builds upon the proven reliability of FEA as an indispensable tool for predicting complex structural behavior, establishing it as the foundational methodology for this work.

The application of these advanced design techniques is paramount given the severe consequences of failure in lifting operations. The operational environment is governed by stringent safety regulations such as OSHA 1926.251^13^, which mandate rigorous inspection, testing, and certification protocols to protect personnel and assets. Furthermore, the integrity of a lifting system is only as strong as its weakest component. Studies on auxiliary equipment highlight critical risks, such as dangerous stress concentrations in non-standard connections^14^ and the complex design challenges of specialized tackle for heavy loads^15^. This holistic view of the lifting system—from the primary spreader beam to its connections and slings—underscores the critical need for a validated, standards-compliant design methodology for all components.

The foundation of safe and reliable design rests upon adherence to codified international standards, which provide the critical link between theoretical analysis and proven practice. The ASME BTH-1 standard, in particular, establishes the essential design criteria for below-the-hook lifting devices. Its development, as documented by Duerr^16^, was a direct response to the industry confusion stemming from the overly simplistic design factor mandate in its predecessor, ASME B30.20, and provides a sophisticated framework for addressing various structural limit states^17^. However, as critically reviewed by Williams^18^, the application of such standards involves complex interpretations of factors of safety and requires careful analysis against statutory regulations. This historical context underscores the necessity for designs to be not only compliant but also critically evaluated against the latest standards and real-world loading conditions. This principle is also emphasized in the design of specialized tooling to meet ASME BTH-1 safety margins^19^.

However, a significant and often overlooked disconnect persists between digital simulation and physical certification, representing a critical industry challenge. While FEA provides powerful predictive capabilities, its true value is only realized when it is part of a closed-loop, iterative process that is rigorously validated by physical testing. Many commercial offerings and practical designs suffer from a lack of transparency in their engineering basis, often relying on either overly conservative (and costly) “over-design” or risky under-engineering validated only by a single proof test. This ad-hoc approach leads to two major inefficiencies: the substantial financial waste associated with material overuse and failed prototypes during testing, and the latent risk of in-service failure due to unvalidated stress concentrations or unaccounted-for fabrication defects. The literature reveals a tendency for studies to focus on isolated aspects—either pure FEA validation^20^ or algorithmic design selection^12^—without providing a comprehensive, start-to-finish protocol that is fully traceable and adherent to international standards from the first CAD model to the final certified product. This gap leaves engineers without a clear, replicable blueprint for developing optimized, safe, and cost-effective custom lifting equipment.

To address this unequivocal gap, this paper presents a novel, holistic engineering workflow for the design, analysis, fabrication, and validation of a 30-ton spreader beam. The primary novelty and contribution of this work is the demonstration of a fully integrated, start-to-finish protocol that moves seamlessly from FEA-based iterative design to a physically validated and certified lifting asset. This end-to-end methodology, rigorously compliant with international standards (ASME BTH-1, OSHA, AWS D1.1), provides a transparent and replicable blueprint that bridges the critical divide between digital simulation and physical certification in industrial practice.

Furthermore, this study serves as a foundational case study and an open invitation for increased academic participation in this critically under-published field. It develops a structured workflow for designing a 30-ton spreader beam that ensures compliance with international safety regulations. By meticulously documenting every stage—from initial concept and iterative FEA analysis to material traceability via MTCs, standardized fabrication, pre- and post-test NDT (PT/MT), and a conclusive 133% SWL proof test—this work provides a transparent, replicable framework. It is intended to bridge the current divide between commercial practice and rigorous engineering science, encouraging further research into practical, innovative solutions that enhance safety and efficiency in heavy lifting operations.

## Methodology (step-by-step workflow)

**Fig. 1:**
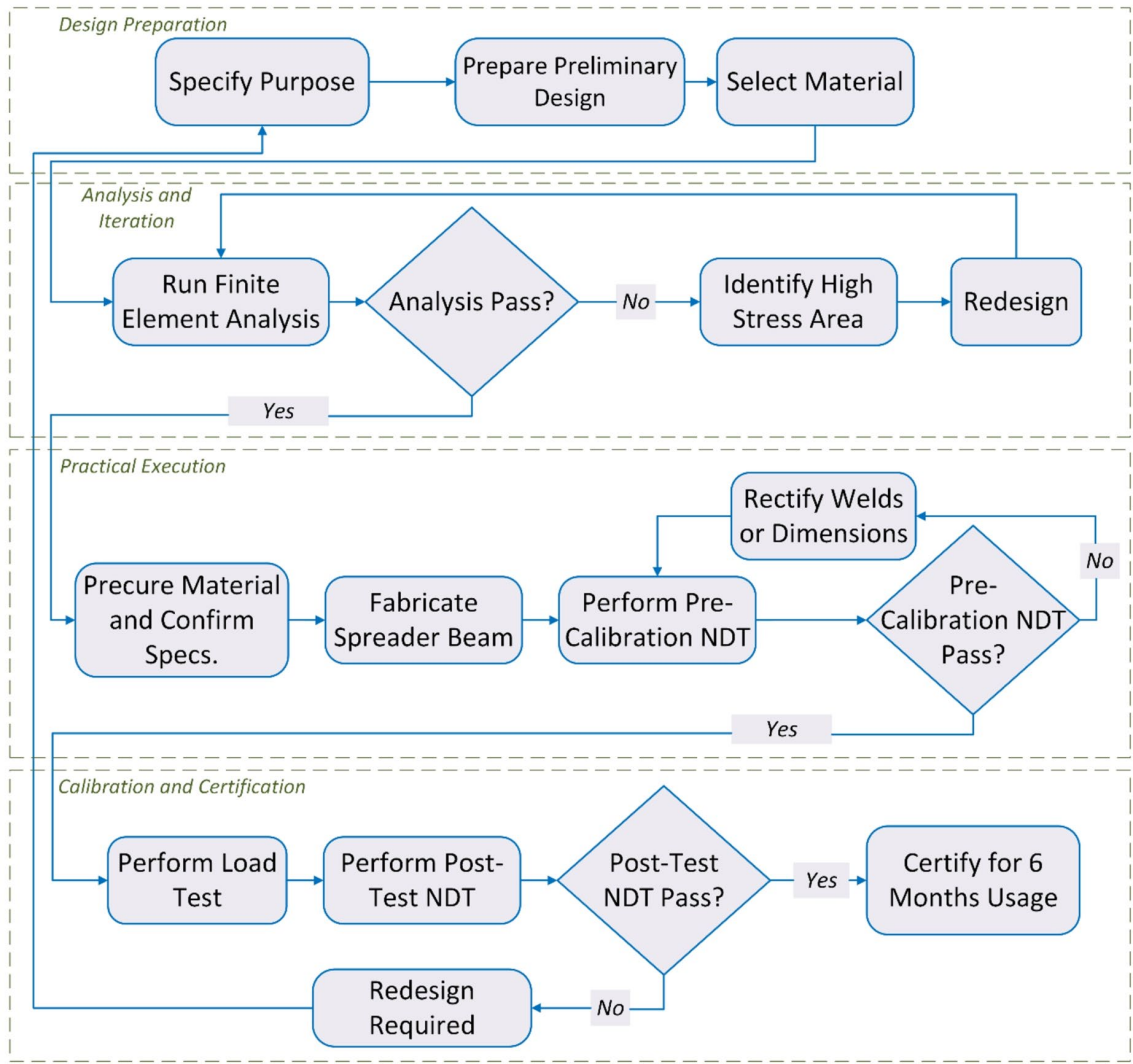
Systematic flowchart of the research methodology for the development of Below-the-Hook Lifting Devices (BTH), as applied to the 30-ton spreader beam.

The methodology of this research is structured to provide a systematic approach to the design, analysis, fabrication, and validation of the 30-ton spreader beam or any Below-the-Hook Lifting Devices (BTH). Figure 1 illustrates this step-by-step process. The process begins with the *design preparation* stage, where the purpose of the beam is defined, an initial design is developed, and the appropriate material is selected. The next stage is *analysis and iteration*, which involves performing Finite Element Analysis (FEA) on the design. If areas of high stress are identified, the design is modified, and the analysis is repeated until an acceptable solution is achieved. The methodology then advances to the *practical execution* stage. This stage involves procuring materials, fabricating the beam, and conducting pre-calibration non-destructive testing (NDT). Should the beam fail this inspection, the welds or dimensions are corrected and re-inspected, repeating until the beam passes. The final stage is *calibration and validation*. The beam undergoes load testing followed by a final round of post-test NDT. A successful test validates and approves the beam design. If it fails at this point, the process loops back, requiring a redesign.

As applied to the present case study, the material selection (2 grades: ASTM A106 Gr. B and ST37/A36) is detailed in *Material Selection* Section and Table 2. The FEA model geometry is the finalized design from the iterative process shown in Fig. 5. Non-destructive testing (PT/MT) was integrated as a critical quality gate, performed pre- and post-test in strict accordance with the AWS D1.1 and standard certification norms, as described in Sections: *Practical Fabrication & Inspection* and *Calibration & Load Testing*.

### Define purpose & requirements

**Fig. 2:**
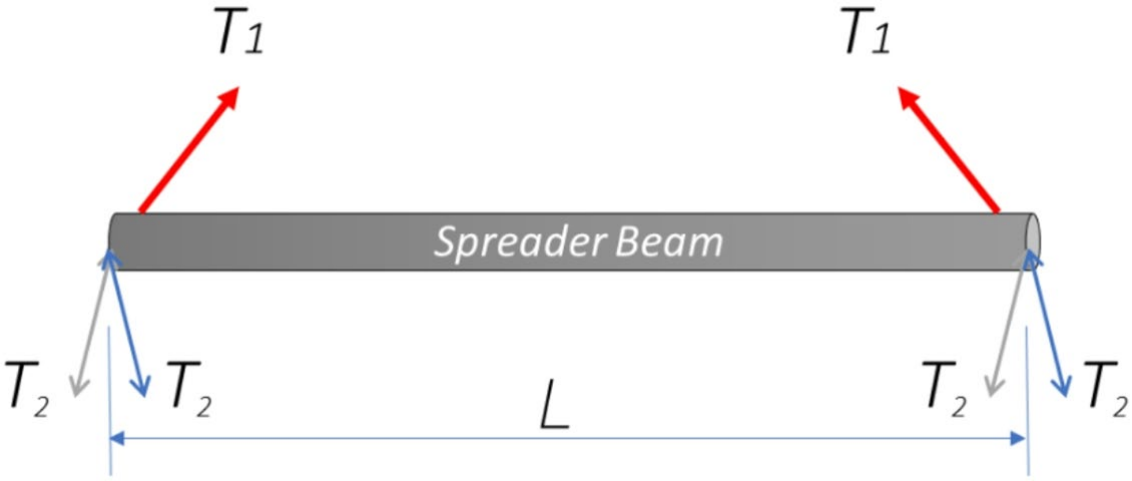
Schematic outlining the primary design requirements for the 30-ton spreader beam case study: a 30-ton SWL with a 2:1 design factor (ASME BTH-1, Category A^1^, a two-top/four-bottom lug configuration, and a defined span length for load clearance.

The foundational phase of designing any lifting appliance is the unequivocal definition of its purpose and operational requirements. This establishes the design constraints and functional criteria that all subsequent decisions must satisfy. For the case study of a 30-ton capacity spreader beam, the primary requirements were defined as follows and shown in Fig. 2: a safe working load (SWL) of 30 metric tons, necessitating a design load incorporating a safety factor of 2:1 as per ASME BTH-1 guidelines for category A Lifters for service class 0^21^; a configuration with two top lugs for connection to the crane hook and four bottom lugs for attaching to the load, two at each side; and a specified span length to ensure stability and clearance for the intended loads. Furthermore, all design calculations, material selection, and fabrication procedures were mandated to comply with relevant international standards, including ASME BTH-1 for design and AWS D1.1 for welding. This clear delineation of capacity, geometry, and regulatory adherence provides the essential framework for the preliminary design phase.

### Preliminary design

The first and crucial element to design, based on the spreader beam outline (Fig. 2), is the main load-bearing element. For this application, a cylindrical section is favorable over standard profiles due to its uniform strength characteristics and high torsional rigidity. The initial feasible step was to select the dimensions and material for this element. Based on empirical experience, the first trial utilized a standard *6-inch nominal diameter* pipe per ASME B36.10 (ASTM A106 Gr. B). The specified geometry had an outer diameter of 168 mm, an inner diameter of 146 mm, and a wall thickness of 22 mm.

The beam was analyzed under a simplified loading condition replicating the final application. A central point load of 30 tons (294 kN) was applied, representing the total weight transferred from the load through the slings to the lifting lugs at each end of the beam. This configuration induces a state of pure bending in the main span. The resulting Von Mises stress and deflection were evaluated using Finite Element Analysis (FEA).

**Fig. 3:**
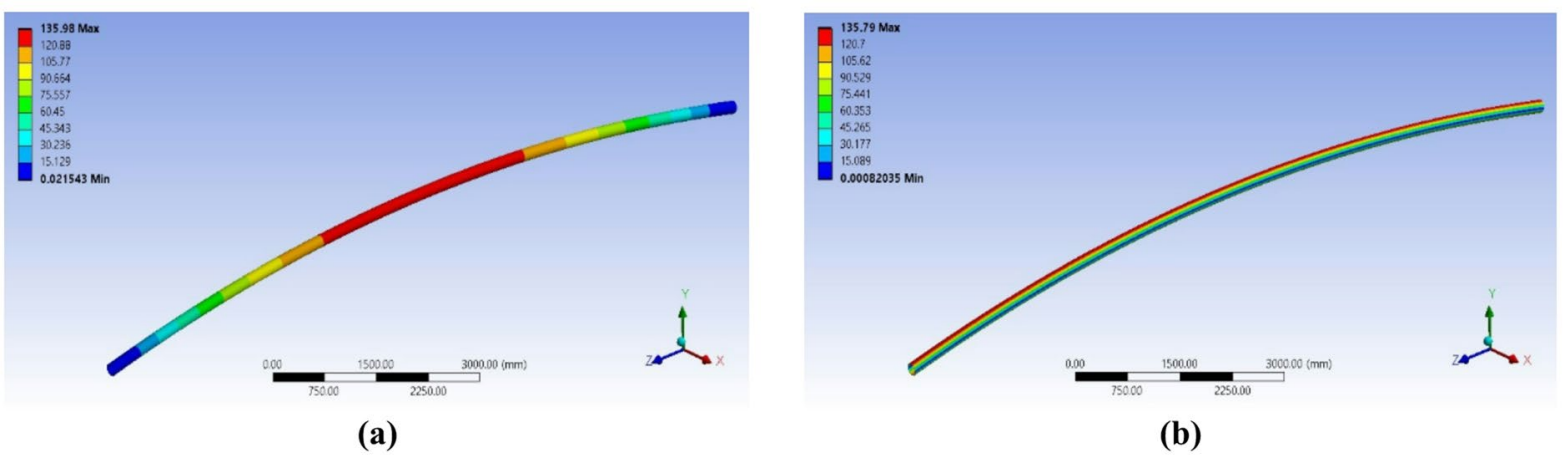
Numerical results from the iterative design process: (**a**) Deformation (**b**) Von Mises stress distribution for the initial 6-inch pipe design under a 30-ton load, *Finite element results generated using ANSYS R18.1*.

The numerical results for this initial design showed a maximum Von Mises stress of *135.7 MPa* and a substantial elongation (deflection) of *136 mm* (Fig. 3). Given the specified minimum yield strength of 240 MPa for ASTM A106 Gr. B material, this resulted in a safety factor of *1.77*, which is below the target value of 2.0. The analytical calculation for bending stress confirmed a close agreement, yielding a value of *135 MPa*, which validated the FEA approach.

**Fig. 4:**
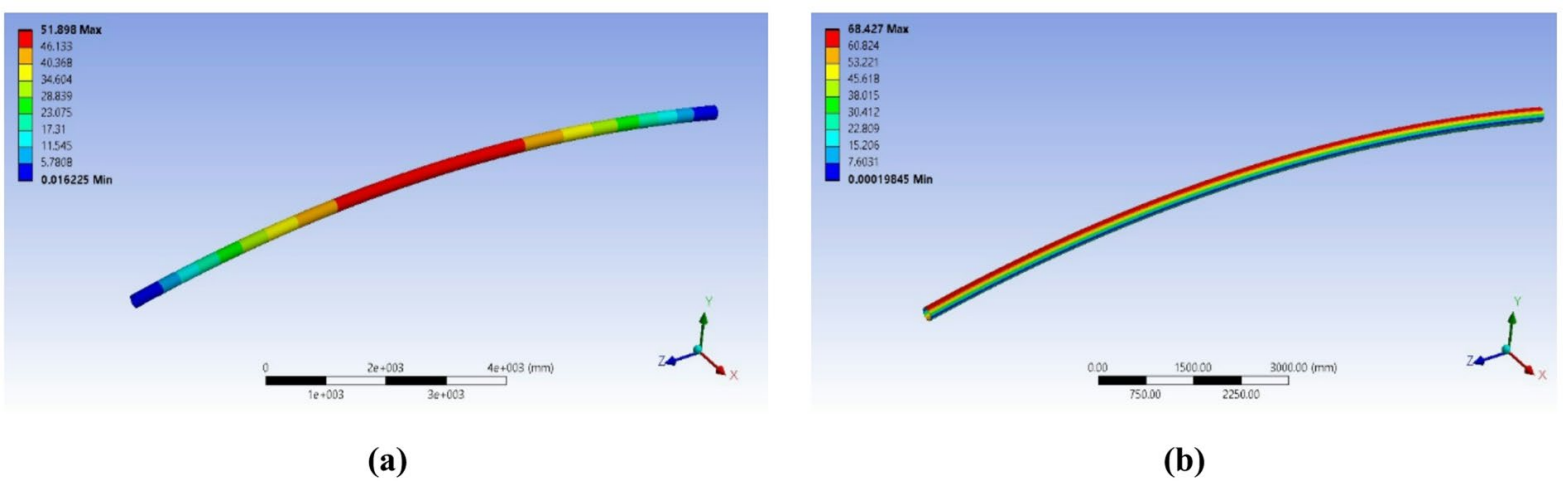
Numerical results from the iterative design process: (**a**) Deformation (**b**) Von Mises stress distribution for the Proposed 8-inch pipe design under a 30-ton load, *Finite element results generated using ANSYS R18.1*.

Based on these findings, a revised section was proposed to meet the safety factor requirement. The new design featured an outer diameter of *219.1 mm*, an inner diameter of *193.7 mm*, and a wall thickness of *12.7 mm*. The simulation results for this configuration showed significantly improved performance. The maximum Von Mises stress was reduced to *68.4 MPa* and the elongation was limited to *51.8 mm* (Fig. 4).

This resulted in a safety factor of 3.51 (240 MPa / 68.4 MPa), comfortably exceeding the target. While stress was a primary driver, a key constraint was limiting elastic deflection to ensure stability and control, a critical consideration for the long (~ 12 m) span when handling precision machinery. It is important to note that while standards like ASME BTH-1 provide strict stress criteria, they do not specify definitive deflection limits, leaving this to engineering judgment. For this project, the substantial deflection of 136 mm in the initial 6-inch design was deemed unacceptable as it could lead to instability. A target deflection was established to prevent this, and the optimized 8-inch design reduced the deflection to a manageable 51.8 mm, successfully meeting this serviceability requirement. Consequently, this second design was adopted for the final spreader beam configuration (Table 1).

**Table 1:** Comparative summary of FEA and analytical results for the initial and optimized beam designs, showing von mises stress, deflection, and calculated safety factor.

Analysis method	FEA result (6-in. design)	FEA result (8-in. design)	
Von Mises Stress, σ_vm_	135.7	68.4	MPa
Mid-Span Deflection, δ	136.0	51.8	mm
Safety Factor (S_y_/σ_vm_)	1.77	3.51	–
Status	Fail (SF < 2.0)	Pass (SF > 2.0)	

**Fig. 5:**
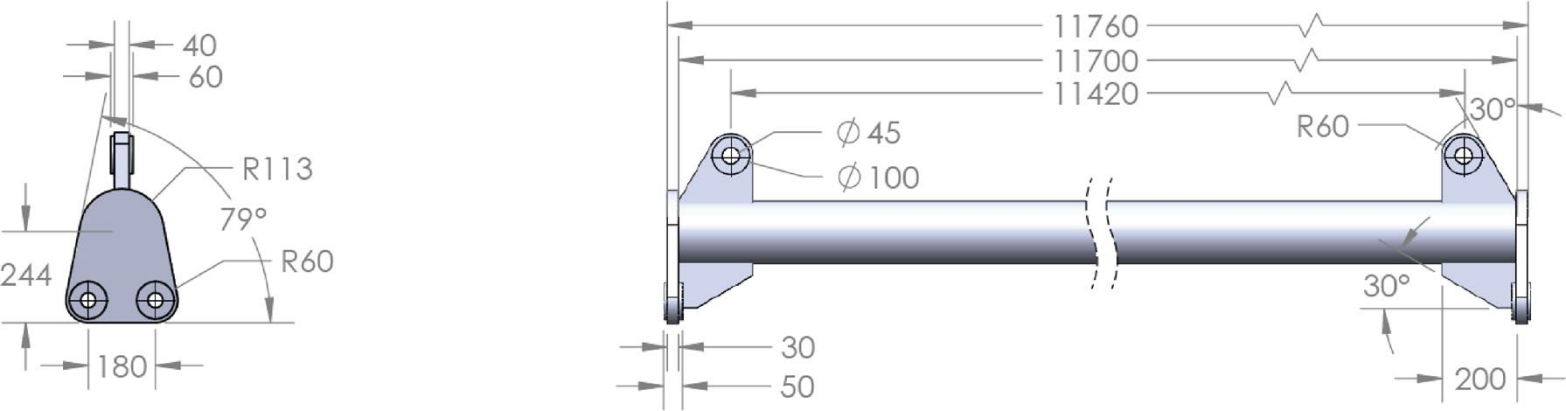
Proposed primary design of the 30-ton spreader beam fabricated from an 8-inch pipe. The configuration features a 3-point connection; a central top lug to connect to the crane hook and two bottom lugs for attaching to the load, as required by the design specifications. All dimensions are in millimeters (mm).

Based on the iterative sizing process that confirmed the 8-inch pipe as the optimal main beam, the primary design was settled to incorporate the functional lifting connections. The settled configuration, illustrated in Fig. 5, integrates the ASTM A106 Gr. B pipe as the main longitudinal body, reinforced with ST37 end plates and welded pad-eyes (lifting lugs). These lugs are equipped with precisely machined holes to accommodate shackle pins and lifting slings, forming the required three-point connection system: a single top connection for the crane hook and two bottom connections for the load. This structural arrangement is designed to ensure a uniform distribution of bending stresses across the beam’s length while minimizing stress concentrations at the critical weldments and lug interfaces. The design is prepared according to engineering standards, providing both safety and reliability during lifting operations. Detailed sectional views and dimensions included in the drawing support accurate manufacturing and quality assurance.

### Material selection

The selection of materials for the spreader beam was driven by three primary factors: *mechanical strength* to safely withstand the design loads, *excellent weldability* to ensure the integrity of fabricated connections, and *commercial availability* to facilitate procurement and minimize cost. The proposed materials, derived from the preliminary design (Fig. 5), satisfy these criteria and are detailed in Table 2. The main load-bearing element is a *Seamless Carbon Steel Pipe per ASTM A106 Grade B*,* Schedule 80*. This material was selected for its high yield strength (240 MPa), proven performance in structural applications, and isotropic properties that provide uniform strength in all directions, which is critical for handling potential off-axis loading conditions. The connection lugs and stiffening plates are fabricated from *hot-rolled carbon steel plates per ASTM A36*. This grade offers a good balance of yield strength (minimum 235 MPa), ductility, and exceptional weldability using common methods. Its widespread use in construction and fabrication ensures easy availability in local markets, making it a cost-effective choice for these components. The compatibility of ASTM A106 Gr. B and A36 in terms of thermal expansion and carbon equivalent value ensures reliable and crack-free welding, which is paramount for the structural integrity of the assembled spreader beam.

**Table 2:** Material selection summary example.

Component	Material standard	Grade	Key properties	Justification
Main Beam	ASTM A106	Grade B, Seamless	Yield Strength: 240 MPa, Tensile Strength: 415 MPa	High strength-to-weight ratio, isotropic properties, excellent for bending loads.
Lugs & Stiffeners	DIN 17,100/ASTM A36	St37/A36	Yield Strength: 235 MPa, Tensile Strength: 360–510 MPa	Excellent weldability, readily available, cost-effective, sufficient strength for connection details.

### Structural analysis and iterative optimization via FEA

The preliminary design was subjected to a detailed structural assessment and optimization process using Finite Element Analysis (FEA). The goal of the iterative optimization was to achieve the safety factor mandated by ASME BTH-1 by minimizing the maximum von Mises stress through geometric modifications. Controlling deflection was a secondary, serviceability objective. This direct, FEA-driven approach is a practical and effective methodology for optimizing custom, safety-critical components for structural integrity. The process to achieve this is detailed in the following subsections.

#### FEA model setup and verification

The finite element analysis was performed using a static structural solver in ANSYS Workbench 18.1. The mathematical model assumed a linear-elastic, isotropic material response based on the minimum specified yield strengths of ASTM A106 Gr. B and ST37. All components (main pipe, lugs, stiffeners) were connected using bonded contact, simulating a full-penetration weld as per the AWS D1.1 fabrication procedure. The geometry was discretized using Solid186 elements (second-order 20-node hexahedral solids), selected for their high accuracy in capturing bending and contact stresses. The boundary conditions simulated a remote displacement constraint on the top lug to represent the crane hook, while the 30-ton (294 kN) load was applied as a bearing force equally distributed across the four bottom lugs, replicating the worst-case static service load.

#### Initial FEA results and identification of failure

**Fig. 6:**
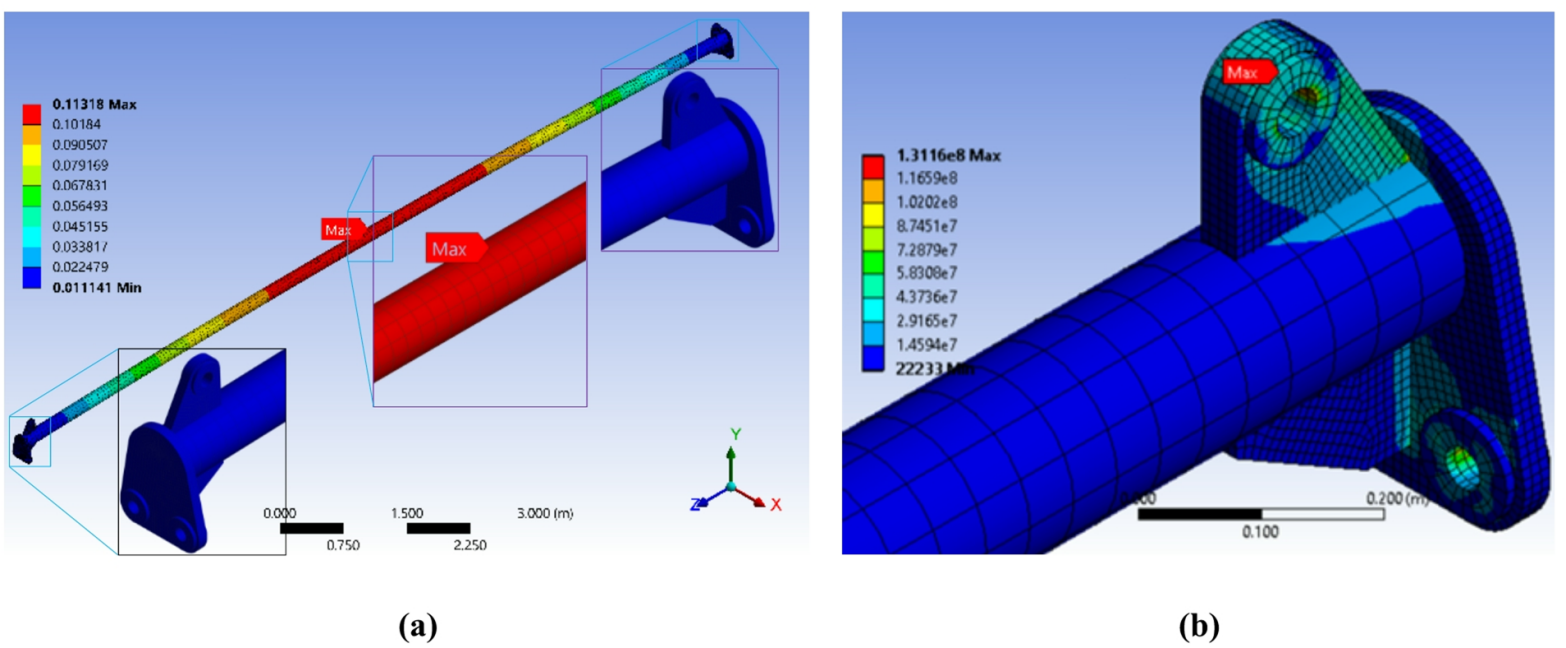
Initial FEA results of the proposed primary design of the 30-ton spreader beam fabricated from an 8-inch pipe, showing: (**a**) the total deformation along the spreader beam and (**b**) a detailed view revealing a critical von Mises stress of 131 MPa at the junction of the main pipe, stiffener, and top plate, *Finite element results generated using ANSYS R18.1*.

The initial FEA results, presented in Fig. 6, revealed critical structural inadequacies. The maximum von Mises stress was approximately *131 MPa*, located at the stress concentration point where the main pipe, stiffener, and top loading plate converge (Fig. 6b). Given the yield strength of the primary material (250 MPa for ASTM A36), this resulted in a safety factor of *1.9*, which is below the minimum required value of 2.0 and confirmed the design was unsafe for its intended service.

#### First design iteration

**Fig. 7:**
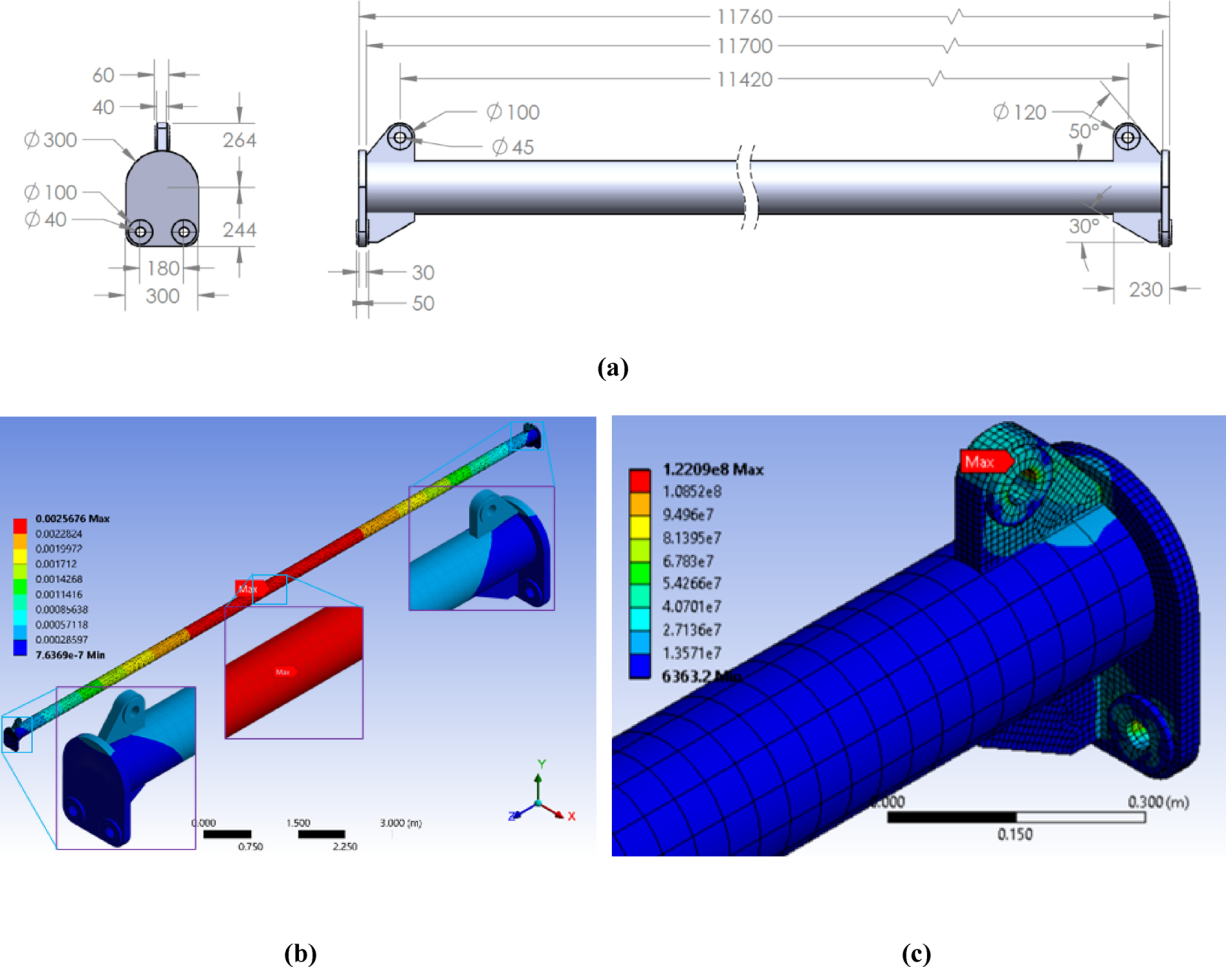
First design iteration results: (**a**) the modified design with reinforced side plates and a smoother load path, (**b**) the total deformation along the spreader beam, and (**c**) the resulting von Mises stress distribution, showing a maximum stress of 122 MPa that has shifted to the bore of the main top lifting lug. All dimensions are in millimeters (mm), *Finite element results generated using ANSYS R18.1*.

To mitigate the high stress concentration, a first design modification was implemented. The dimensions of the side lifting connection plates were increased, and the geometry of the connection between the top lug plate and the side plates was redesigned to provide a smoother load path (Fig. 7a). The analysis of this revised model under identical loading conditions showed a significant improvement, with the maximum von Mises stress reduced to *122 MPa* (Fig. 7c). However, the corresponding safety factor of 2.05 was slightly above the required threshold. The stress concentration point had also shifted to the hole of the main top lifting lug, primarily due to shear stress.

#### Second design iteration and final optimization

**Fig. 8:**
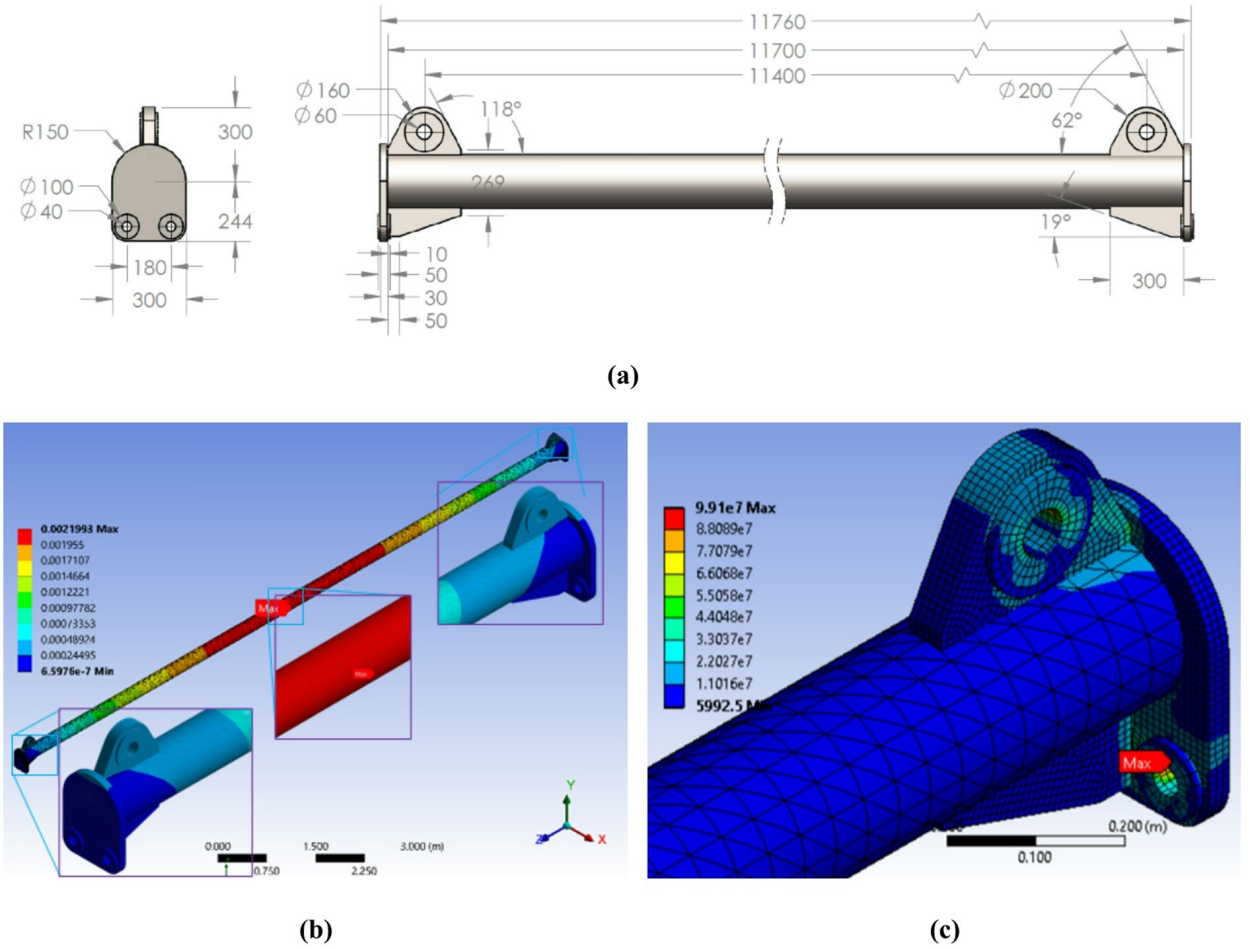
The final, manufacturable design: (**a**) key modification showing the reinforced top lug assembly to enhance shear resistance, (**b**) the total deformation along the spreader beam, and (**c**) the results of the converged FEA model, showing a uniform stress distribution with a maximum von Mises stress of 99.1 MPa. This results in a safety factor of 2.52, successfully validating the structural integrity for the 30-ton service load. All dimensions are in millimeters (mm), *Finite element results generated using ANSYS R18.1*.

A second design iteration was conducted to address the remaining stress concentration identified in the previous analysis. As shown in Fig. 7(c), the locus of maximum stress had shifted to the bore of the main top lifting lug, a phenomenon primarily attributed to shear and bearing stresses induced by the pin connection. To mitigate this, the design was enhanced by reinforcing the top lug assembly, effectively increasing the cross-sectional area subjected to shear and improving the load distribution around the pin hole (Fig. 8a). This modified configuration was analyzed and subsequently refined through a final iterative process to achieve an optimal stress distribution.

The final optimized configuration, shown in Fig. 8b, was employed using a static structural solver with a model consisting of approximately 158,000 Solid186 elements (second-order 20-node hexahedral solids) and 235,000 nodes, capable of accurately capturing bending and contact stresses. Mesh quality was evaluated through aspect ratio, skewness, and Jacobian metrics, all maintained within ANSYS-recommended limits (skewness < 0.85), with local refinement applied around lug holes and weld intersections. Mesh convergence was verified by ensuring that the maximum von Mises stress changed by less than 2% upon further refinement. The FEA results confirmed a maximum von Mises stress of 99.1 MPa. Based on the material’s yield strength (250 MPa for ASTM A36), this resulted in a safety factor of 2.52, exceeding the minimum requirement of 2.0 stipulated by ASME BTH-1. Deformations were within acceptable limits, and the stress distribution was uniform, exhibiting no critical concentrations. This confirms the beam’s structural integrity and its ability to handle the 30-ton load safely. Consequently, this design was approved for manufacturing, forming the basis for the prototyping and experimental validation phases detailed in the following sections.

### Practical fabrication & inspection

The optimized design entered the fabrication and inspection phase to ensure the physical assembly conformed to design intent and quality standards. All materials, as specified in the *Material Selection* section, were procured. Their corresponding Mill Test Certificates (MTCs) were rigorously verified to ensure compliance with the stipulated chemical and mechanical properties.

**Fig. 9:**
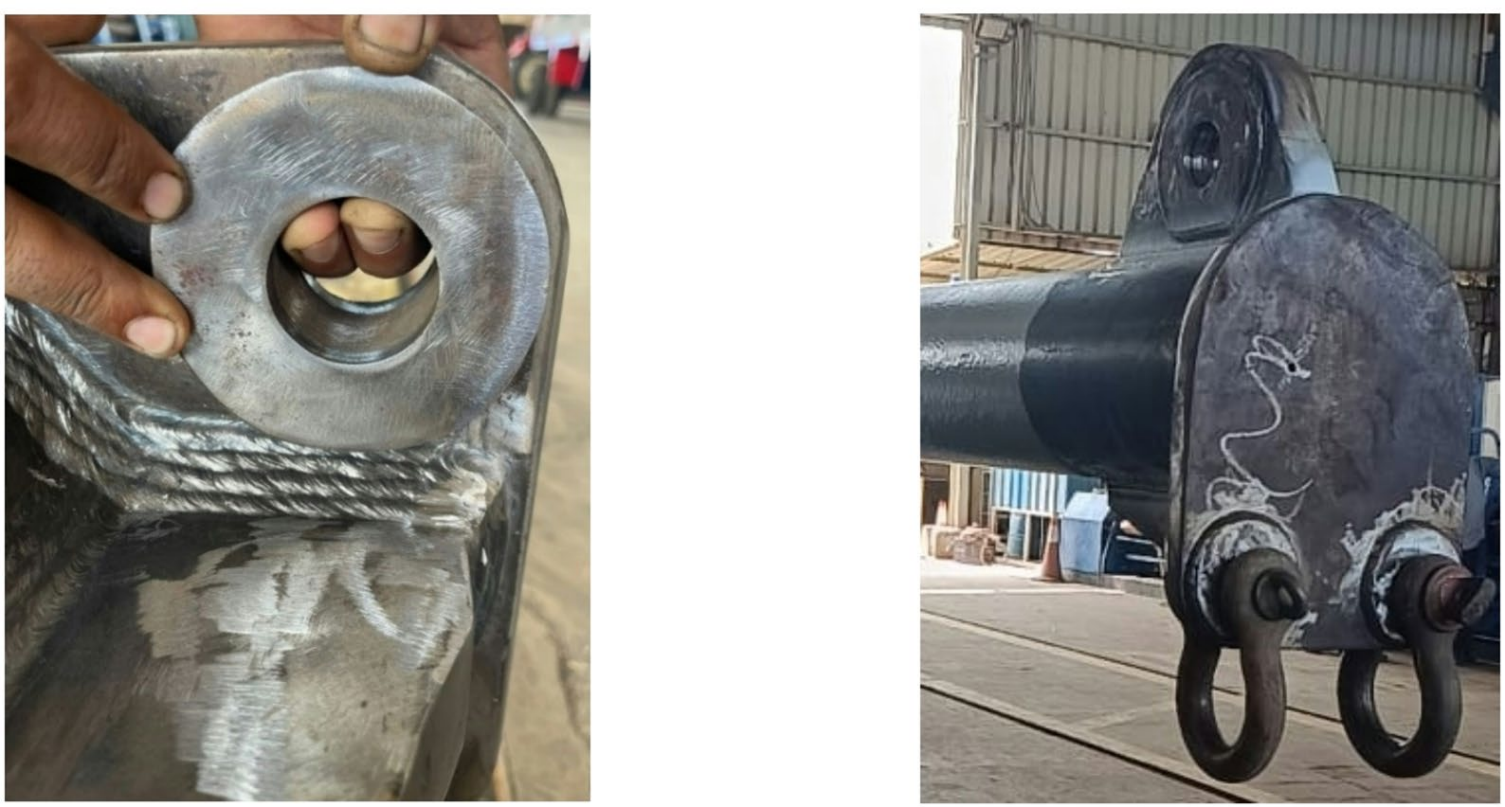
Fabrication and welding of the beam assembly by certified personnel, adhering to AWS D1.1 standards.

Fabrication was executed under strict quality control protocols. All welding adhered to qualified AWS D1.1 procedures performed by certified welders (Fig. 9). While the current FEA model did not simulate thermal effects from welding, their impact (e.g., residual stress and heat-affected zone damage) was mitigated and controlled through these strict procedural adherence. Dimensional conformance to the final design drawings was verified at critical assembly stages, ensuring geometric accuracy of lug alignment and spacings.

**Fig. 10:**
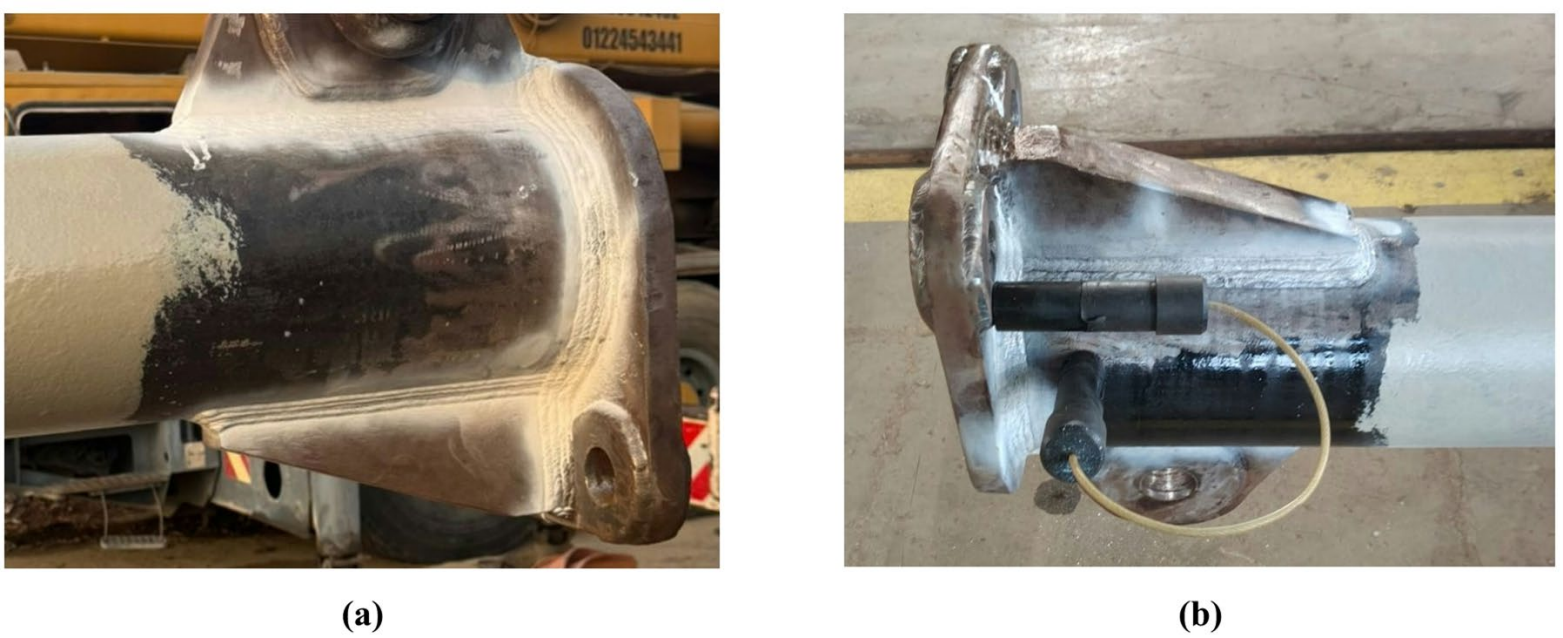
Non-destructive testing (NDT) of the 30-ton spreader beam weldments. (**a**) Liquid Penetrant Testing (PT) indication of a surface-breaking defect at a critical weld toe. (**b**) Magnetic Particle Testing (MT) revealing a near-surface flaw in a ferromagnetic lug component, ensuring structural integrity for lifting applications.

Upon welding completion, a comprehensive Pre-Calibration Non-Destructive Testing (NDT) regime was implemented to qualify the prototype. *Liquid Penetrant Testing (PT)* was applied to all accessible welds and critical areas to detect surface-breaking defects (Fig. 10a). *Magnetic Particle Testing (MT)* was used on ferromagnetic components as a redundant method for identifying surface and near-surface flaws. This NDT was specifically employed to detect any defects, such as cracks or lack of fusion, that could have been initiated by the manufacturing process, thereby ensuring structural integrity prior to proof load testing (Fig. 10b).

### Calibration & load testing

**Fig. 11:**
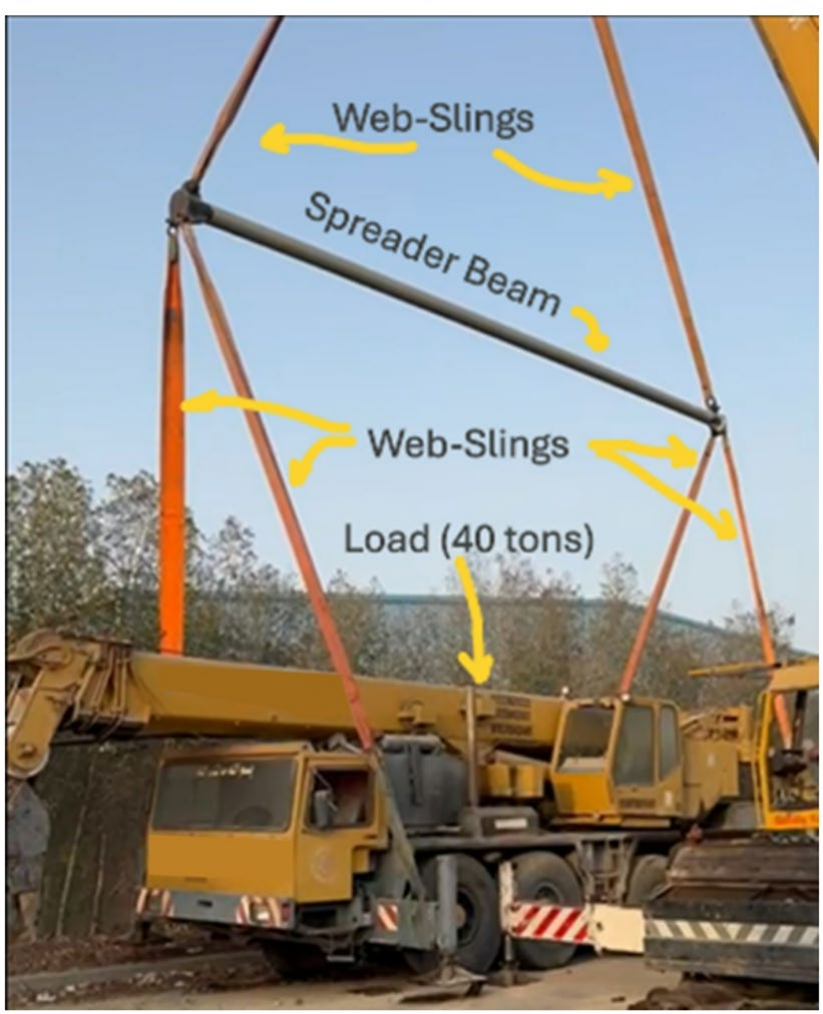
Setup for the 40-ton proof test in a controlled area. All rigging equipment (slings, shackles) was inspected and had a Working Load Limit (WLL) exceeding the test load.

The final and most critical phase in the validation of the 30-ton spreader beam was physical calibration and load testing. This process serves as the ultimate verification of the design calculations, fabrication quality, and structural integrity, ensuring the beam performs safely under its rated load and beyond. The procedure was conducted in strict accordance with international standards, including OSHA 1926.251(a)(4) for proof-testing and ASME BTH-1 (Category A, Service Class 0), which mandate a proof test 125% of the Safe Working Load (SWL). For this 30-ton (SWL) beam, a pre-weighted 40-ton load (133% of SWL) was utilized for the test (Fig. 11).

#### Test setup and safety protocol

A comprehensive safety plan was enacted in a controlled, isolated area. All personnel were qualified and trained in test and emergency procedures. Auxiliary lifting gear, inspected by a competent person pre-test, included, as illustrated in Fig. 11:

A *crane* certified for a capacity exceeding the total test weight (40 tons plus the weight of the spreader beam and rigging).

- A certified 40-ton *pre-weighted mass*, serving as the calibrated test load.
- *Web slings* with a Working Load Limit (WLL) rated for the test load, marked in accordance with OSHA standards.
- *Shackles and hooks* with a rated capacity at least equal to that of the slings.

#### Test execution and monitoring

The test load applied *slowly and gradually* to avoid dynamic shock loading, which is prohibited by safety standards. The load was held at the full 40-ton capacity for a minimum duration of *5 to 10 min*. During this hold period, the beam monitored for:

- *Audible indications* of stress, such as cracking or creaking.
- *Visual signs* of permanent deformation or yielding in any structural member.
- *Movement or distortion* at welding points and lug connections.
- Deflection gauges should be used, if available, to measure and record any deformation.

#### Post-test non-destructive testing (NDT)

Upon successful completion of the load test and subsequent unloading, the beam underwent a final *Post-Test NDT* inspection. This is crucial for detecting any micro-cracks or defects that may have been initiated by the proof load but are not visible to the naked eye. The primary method for inspecting critical welds and high-stress areas was *Magnetic Particle Testing (MT)*. MT is highly sensitive for detecting surface and near-surface flaws in ferromagnetic materials like carbon steel and is a required step for ensuring the integrity of the beam after testing. This was complemented by *Liquid Penetrant Testing (PT)* for further validation of surface cracks.

#### Certification and recertification cycle

After passing the 40-ton test and NDT without deformation or defects, a certificate of proof test was issued. The beam is certified for use for 6 months, after which it must be reinspected and recertified.

#### Failure mode and iterative redesign

Should the beam exhibit permanent deformation, fail the load test, or should the post-test NDT reveal critical defects, the process enters a failure investigation mode. This indicates a potential flaw either in the *design assumptions* (e.g., underestimated dynamic factors, stress concentrations) or in the *fabrication quality* (e.g., weld defects). The beam must be immediately taken out of service. The entire process, from design review (*Define Purpose & Requirements* Section) to FEA analysis (*Structural Analysis an Iterative Optimization via FEA* Section) and fabrication (*Practical Fabrication & Inspection* Section), must be reassessed. The location of the failure or high stress informs the necessary modifications, such as adding stiffeners, increasing material thickness, or improving weld procedures, before the iterative process begins anew.

## Discussion

The primary contribution of this work is not merely the design of a single spreader beam, but the demonstration and validation of a holistic, integrated methodology that systematically bridges the gap between digital simulation and physical certification for below-the-hook lifting devices. This start-to-finish protocol—seamlessly linking iterative FEA-driven design with standards-compliant fabrication, NDT, and a formal proof test—addresses a critical need in industrial practice that is seldom documented in the scientific literature with such completeness.

The successful outcome of this process provides compelling validation of the proposed framework. The finite element model, whose accuracy was a cornerstone of the design process, demonstrated strong qualitative agreement with the physical test. More significantly, the device’s successful certification under a static proof load of 133% of its SWL serves as the ultimate, industry-recognized validation of the entire methodology. This passing test result confirms that the predictions of the FEA model were not just academically interesting but were practically accurate enough to yield a safe, certifiable, and field-ready asset.

It is important to acknowledge the limitations of this study to contextualize its findings and guide future work. The most notable limitation was the absence of quantitative strain gauge data during the proof test, which would have provided a direct, point-by-point comparison with the FEA results. While the successful certification validates the global structural adequacy, future research could incorporate such instrumentation to further refine the correlation between local FEA predictions and measured strains, particularly in complex, multi-axial stress regions like the lug holes.

**Fig. 12:**
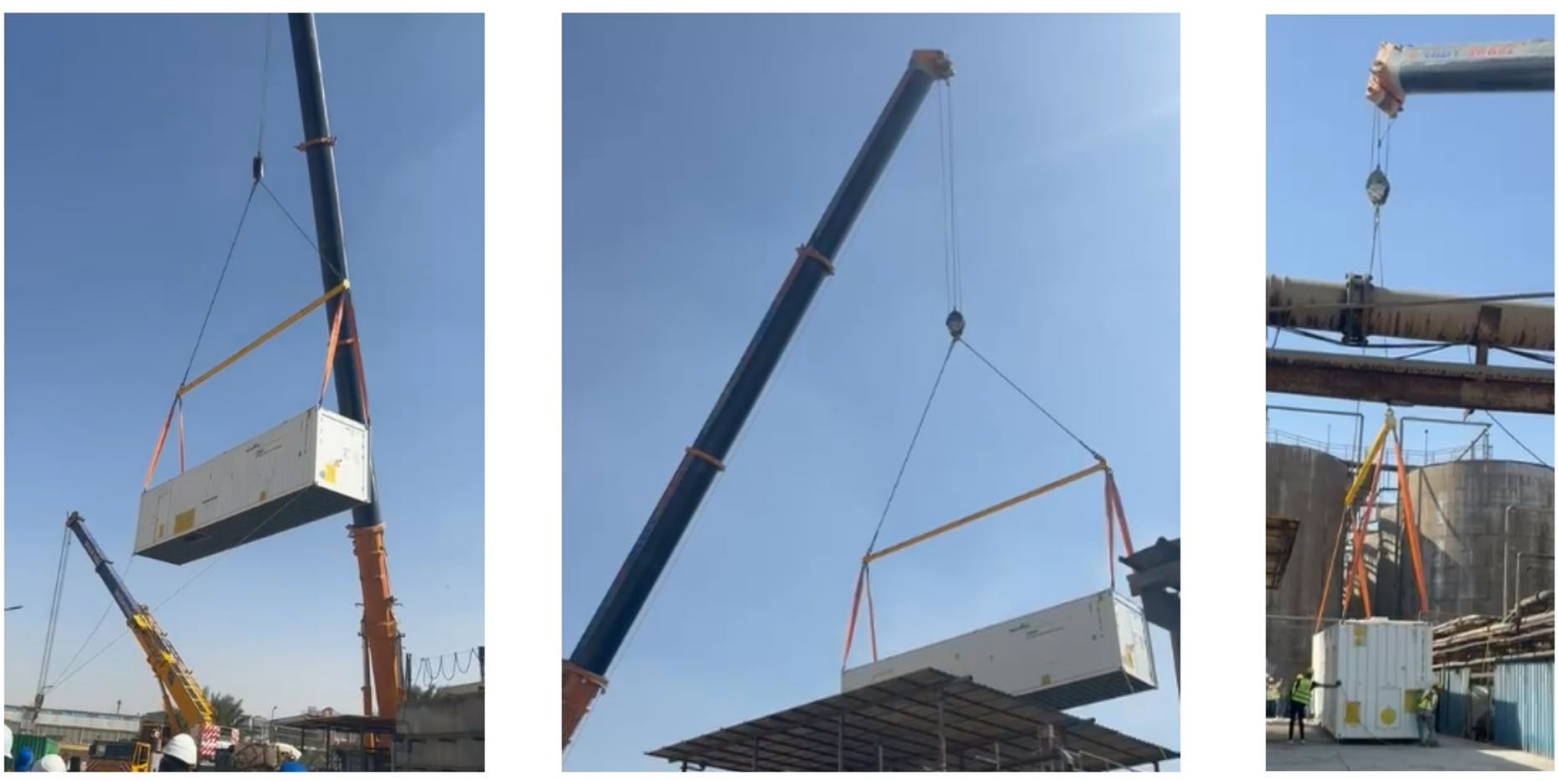
Final deployment of the certified 30 ton spreader beam successfully lifts the equipped container (23-ton net load) to its final position.

This paper has presented a replicable, step-by-step engineering workflow that is both rigorous and practical. By demonstrating the entire journey from a concept validated by FEA to a physically certified lifting device, this work provides a valuable template for engineers and researchers. The methodology proves that a disciplined integration of computational analysis with established fabrication and validation standards is a powerful and reliable path for designing critical load-handling equipment, ensuring both structural performance and regulatory compliance (Fig. 12).

## Conclusion

This study successfully detailed the end-to-end engineering process for a 30-ton spreader beam, from initial concept to a certified lifting asset. The integrated methodology combining FEA-driven design iteration, standards-compliant fabrication (AWS D1.1), and rigorous physical validation (OSHA, ASME) proved to be robust and effective. The final design, based on an 8-inch ASTM A106 Grade B pipe and ST37 lugs, achieved all structural performance criteria, with a safety factor exceeding 2.0 and successful passage of a 40-ton proof load test. The project underscores the non-negotiable requirement of physical testing to validate analytical models and ensure absolute operational safety.

### Recommendations

Based on the findings of this study, the following recommendations are proposed:

- Iterative Design: Always employ an iterative FEA process to identify and mitigate stress concentrations before fabrication, as initial designs often require optimization.
- Material Verification: Mandate the review of Mill Test Certificates (MTCs) for all raw materials to ensure chemical and mechanical properties meet design specifications.
- Redundant NDT: Implement a combination of NDT methods (e.g., PT and MT) pre- and post-load testing to detect surface and near-surface defects that could lead to catastrophic failure.
- Conservative Testing: Consider proof testing beyond the minimum standard requirements (e.g., 133% instead of 125% SWL) to provide an additional margin of safety and confidence in the design.
- Digital Workflow: Maintain a digital thread connecting the 3D CAD model, FEA simulations, and fabrication drawings to ensure accuracy and avoid discrepancies during manufacturing.

### For future work

Subsequent studies could explore the integration of topology optimization for weight reduction, the effect of dynamic loading on fatigue life, and the development of automated design tools based on this established workflow.

## Data Availability

The datasets used and/or analyzed during the current study are available from the corresponding author on reasonable request.
